# Supplementary material for: Incarceration history and ethnic bias in hiring perceptions: An experimental test of intersectional bias & psychological mechanisms
Source: PLoS One. 2023 Jan 17;18(1):e0280397. doi: 10.1371/journal.pone.0280397 (PMC9844837; doi:10.1371/journal.pone.0280397)
Supplement: S1 Appendix — (DOCX) [file pone.0280397.s001.docx]

# Appendix A - Participant Instructions

Study Overview

Thanks for taking some time out of your busy schedule to help us learn more about the hiring process. We want you to imagine yourself as part of a hiring committee for a Human Resources Manager position at the University of Washington’s Tacoma campus. The university values external professionals’ views, so they want you to help them decide who to include on their short list. The university is asking the hiring committee to answer very specific questions about each candidate. As a member of this committee, you’d be reading the job ad, reviewing materials from applicants including a cover letter, a resume, and a summary of an interview with the applicant. You will then be answering some questions to let other committee members know what you think about each applicant.

We ask that you please take as much time with these materials as you would when making a real-world hiring decision. If you complete the study too quickly or answer in ways that indicate you’re not paying attention, we likely won’t be able to use your data. We also suggest taking your time and taking notes, so that you can give accurate answers in the questionnaire. Please do the best to answer the questions given the information that you do have.

Some questions we ask will be personal in nature. However, your responses are entirely confidential and will not be linked to any identifiable information (e.g., name, email, etc.).

Thanks again for supporting our students,

**
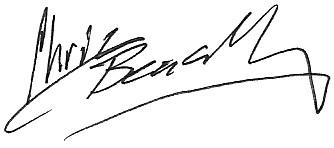
**

## Chris Beasley, Ph.D. and Y. Jenny Xiao
