# Supplementary material for: Incarceration history and ethnic bias in hiring perceptions: An experimental test of intersectional bias & psychological mechanisms
Source: PLoS One. 2023 Jan 17;18(1):e0280397. doi: 10.1371/journal.pone.0280397 (PMC9844837; doi:10.1371/journal.pone.0280397)
Supplement: S2 Appendix — (DOCX) [file pone.0280397.s002.docx]

# Appendix B - Job Ad

Job Advertisement

**Human Resource Manager**

**University of Washington Tacoma**

**Position Summary**

The HR Manager reports to the Assistant Vice Provost of Administrative Services and supervises the UW Tacoma Human Resources team. The HRM is a subject-matter expert responsible for substantive knowledge of human resource guidelines pertaining to Professional and Classified Staff, including adherence to collective bargaining agreements, University policies, and state and local laws. Success in this role requires exercising discretion in highly sensitive personnel matters and building trust within the organization.

**Position Responsibilities**

- Process disability accommodations and FMLA leave requests in accordance with University policy.
- Approver in Workday for Payroll actions
- Create position descriptions for new roles
- Supervision of HR Assistant, as well as Recruiter
- Other duties as assigned

**Position Roles**

- HR Administration (25%)
  - Maintain up-to-date procedures and guidelines for our HR functions.
  - Collaborate with assistant vice provost and campus HR Consultant to provide employee relations support for internal staff.
  - Provide internal training opportunities for staff and management.
  - Analyze new and significantly updated job descriptions for appropriate job classification and exemption status.
  - Process disability accommodations and FMLA leave requests in accordance with University policy.
  - Utilize Workday on a regular basis.
  - Hold security roles such as I-9 coordinator, time and absence approver/initiate, HR Partner, HCM 2, and others.
- Supervision (20%)
  - Embrace and encourage a service-oriented culture across Human Resources
  - Provide staff with clear goals and timelines, and remove obstacles to ensure projects are completed on-time
  - Achieve results through others by demonstrating formal and influence-based leadership
  - Meet frequently with staff to enable communication and ensure day-to-day operations and project-based work are progressing as planned
  - Train and delegate work; require cross training, procedural documentation, and internal auditing; and prevent single points of failure within HR
  - Ensure accuracy of job descriptions, hire, onboard, and mentor staff, provide opportunities for continued learning and skill development, complete performance appraisals, and recommend staff separations
- HR Leadership (20%)
  - Advise staff on best practices to ensure compliance with internal and University-level policies and procedures.
  - Pursue strategies to create a positive, professional, and respectful culture reflects UW values, promotes staff retention, and makes UW a great place to work.
  - Review department practices and policies to ensure compliance with the collective bargaining agreement and University policies.
- Communication (20%)
  - Maintain HR communications and resources to ensure we are providing accurate information on our WIKI page, the T-Drive, and template communication emails.
  - Develop correspondence relating to HR topics.
  - Liaise with Compensation Office, the University Complaint and Resolution Office, Attorney General’s Office, Labor Relations, Safe Campus, and other administrative offices about human resources issues as appropriate.
  - Collaborate with campus and department staff members on the creation of new policies and procedures, the impact of proposed changes and the development of best practices.
  - Provide exit interviews.

**Position Requirements**

- Bachelor’s degree in business, arts and sciences, or related field and 2 years of human resources experience to include
- Compliance with state and federal policies and employment law, to include EEO, FMLA, ADA, FLSA, etc.
- Best practices for performance management
- Employee relations
- Excellence interpersonal skills
- Understanding of payroll procedures

**Preferred Qualifications**

- Master’s degree and
- 4+ years of work experience HR generalist role
- Coaching and training experience
- UW HR experience
- Workday and UWHires experience
- Human resources certification (e.g., PHR, SPHR)

**Benefits offered**:
Job Type: Full-time
Salary: $65,000.00 to $90,000.00 /year
