# Supplementary material for: Incarceration history and ethnic bias in hiring perceptions: An experimental test of intersectional bias & psychological mechanisms
Source: PLoS One. 2023 Jan 17;18(1):e0280397. doi: 10.1371/journal.pone.0280397 (PMC9844837; doi:10.1371/journal.pone.0280397)
Supplement: S3 Appendix — (DOCX) [file pone.0280397.s003.docx]

# Appendix C – Job Applicant Materials

Black Formerly Incarcerated Applicant Cover Letter

JAMAL JACKSON

2345 Aspen Lane; Louisville, KY 40041

Phone: 803.522.9876 * JamalJackson@gmail.com

January 23, 2021

Dear University of Washington Tacoma HR Manager Search Committee,

Your job posting for a Human Resources manager caught my attention because my HR management experience has been in a similar industry, so I am familiar with the challenges. My three years in HR as an assistant and nearly a year as a manager, have allowed me to grow and develop professionally and as a leader. In addition to leading the HR department, I am a strategic planning partner in my current position and serve on the Chancellor’s cabinet.

You are seeking an individual with a strong passion to innovate and drive for solutions. Since I re-imagined the HR department in my current organization and have led to the revision of all people processes, systems, policies, and procedures, I qualify. Personal accountability for results and my integrity are respected and unquestioned. I established a performance development and career planning process that utilizes both internal and external development opportunities for employees including mentoring, job-shadowing, team leadership, and training sessions.

In addition to this professional experience, I am an active member of the Formerly Incarcerated Black Future Professionals Network, which I joined after my release from prison 10 years ago. My proven ability to work effectively with a team has helped us consistently produce strong results with a prominent degree of integrity, dedication, and global vision.

I am most eager to join an organization where HR is respected and where I can continue to contribute to strategic planning issues implementing forward-thinking HR and talent management strategies. Your advertised position appears to fit my experience, accomplishments, and education. I have both HRIS and SHRM-SCP certification.

I will be in your city regularly over the next few weeks and would like the chance to interview with your team and get to know your team. From everything that I can see as an applicant, we are potentially a solid team.

Sincerely,

# Jamal Jackson

# Black Formerly Incarcerated Applicant Resume

**Jamal Jackson**

2345 Aspen Lane

Louisville, KY 84765

Phone: 803.522.9876

Email: JamalJackson@gmail.com

**Summary of Qualifications**

Professional, people-first HR manager with four years in human resources. HRIS certification, and SHRM-SCP certified. Looking to utilize expertise with organizations of 150+ people to manage the HR department at a growing university.

**Work Experience**

Human Resources Manager

Central Kentucky State University

October 2020 - Present

- Key Qualifications & Responsibilities
  - Oversaw a human resources department of 2 team members and their various functions.
  - Effectively liaised between senior management and employees to maintain and improve company-employee relations.
  - Researched, recruited, staffed, onboarded, and trained new company hires according to the needs of department managers and company budget.
  - Ensured compliance of company directives, regulatory concerns, and health and safety protocols.
  - Administered payroll, company benefits packages, corporate events, and team building meetings and outings.
- Key Achievements
  - Implemented a new onboarding process which cut down training times by 4 hours.

Human Resources Specialist

Florida State University

November 2017 - September 2020

- Key Qualifications & Responsibilities
  - Prepared and updated employment records, including pension plans, compensation packages, benefits, disciplinary behavior, and disputes.
  - Administered and processed paperwork related to new hires, job candidates, employment concerns and complaints, and pre-employment tests.
  - Mentored new recruits, provided onboarding seminars, and conducted group and individual training sessions.

**Education**

- MS in Human Resource Management
  Concordia University
  2017
- BS in Business Administration (Human Resource Specialty)
  The University of Florida
  2014

**Volunteer Leadership Experience**

- Formerly Incarcerated Black Future Professionals Network

Members & Secretary

January 2011 - 2014

#

Black Formerly Incarcerated Applicant Interview

1. Tell me a little bit about yourself and what made you consider HR as a profession?
   1. Background:
      1. Growing up, I always heard my family members talk about how hard it was for Black people to find a job. Once I was old enough to start working, I similarly found it difficult and started to wonder about why and what could be done about it. As you’ll see in my background check, my life started to go in a different direction though. I did some things I wish I hadn’t and served time in prison for it. After release from prison, I noticed that a lot of formerly incarcerated people were having trouble getting jobs. The experience reignited my interest in understanding why people struggle finding jobs, and what could be done about it.
   2. College Experience
      1. I received a B.S. in Business Administration from The University of Florida. While I was at the University of Florida, I was a founding member of the Formerly Incarcerated Black Future Professionals Network. I then went on to get an M.S. in Human Resource Management from Concordia.
   3. Work Experience
      1. I started working in the field about six months after graduation and have now been in HR for about four years, as both a specialist and a manager. All of this experience has been in an academic setting. I also hold HRIS and SHRM-SCP certifications. I’m currently hoping to move out West and join a growing university.
2. What do you like most about working in human resources?
   1. I really like the idea of trying to help other managers find the best people for the job as well as make sure people have the resources they need to thrive.
3. Can you tell me about your commitment to and experience with diversity, equity, and inclusion?
   1. As someone who is Black, I have grown up experiencing many instances of prejudice and discrimination first hand. Because of my personal experience, I am deeply committed to promoting diversity, equity, and inclusion. This is an area I’ve devoted a lot of effort in, at my current and previous positions, and I believe it is especially crucial to create awareness among HR professionals, as we could serve as an important gateway that could lead to a diverse organization.
4. Tell me about a challenge you have overcome in life or the workplace and how you overcame it.
   1. By far the biggest challenge I’ve ever faced was coming home from prison. I had been locked up for a while and wasn’t used to shopping or driving, and I was really behind on technology. It was even more challenging, because I had moved from a primarily Black community to one that was mostly White. However, I leaned on my support system to get up to speed. I visited a local reentry center that had technology classes and even a driving refresher class. They also taught me how to limit the choices I was making until I could get used to that again. The center connected me to a local community center that had support groups for Black, Indgenous, and other People of Color who were new to the community.
5. Why do you think you are the best fit for the HR manager position in our company?
   1. I seem to have everything you’re looking for. I have the level of education you prefer, additional certifications, and nearly four years of experience in an academic setting. I’m also someone with a lifelong interest in HR.
6. What do you see as a major event, trend or change that will change human resources in the next five years?
   1. COVID has had a profound impact on our work lives. Many people are itching to get back to work in person, but they haven’t been there for a while, so HR’s task will be to help them all get back to campus seamlessly. Some might not want to work from campus, so we’ll likely need to replace a lot of people too.
7. What questions do you have about this job and/or our company?
   1. Can you share more about the day-to-day responsibilities of this role? How would you describe the pace of a typical day?
   2. What are performance reviews like here and when do they occur?
   3. What would unacceptable, acceptable, good, and excellent performance look like in this role here?
   4. Can you talk about company culture outside of management?
   5. What is the vacation package like?
   6. What are the challenges and/or goals the university is most focused on right now?
   7. Are there any qualifications that you think I'm missing or other concerns about my candidacy?

Latinx Formerly Incarcerated Applicant Cover Letter

JOSÉ LUIS RODRÍGUEZ

2345 Aspen Lane; Louisville, KY 40041

Phone: 803.522.9876 * JoséLuisRodríguez@gmail.com

January 23, 2021

Dear University of Washington Tacoma HR Manager Search Committee

Your job posting for a Human Resources manager caught my attention because my HR management experience has been in a similar industry, so I am familiar with the challenges. My three years in HR as an assistant and nearly a year as a manager, have allowed me to grow and develop professionally and as a leader. In addition to leading the HR department, I am a strategic planning partner in my current position and serve on the Chancellor’s cabinet.

You are seeking an individual with a strong passion to innovate and drive for solutions. Since I re-imagined the HR department in my current organization and have led to the revision of all people processes, systems, policies, and procedures, I qualify. Personal accountability for results and my integrity are respected and unquestioned. I established a performance development and career planning process that utilizes both internal and external development opportunities for employees including mentoring, job-shadowing, team leadership, and training sessions.

In addition to this professional experience, I am an active member of the Formerly Incarcerated Mexican-American Future Professionals Network, which I joined after my release from prison 10 years ago. My proven ability to work effectively with a team has helped us consistently produce strong results with a prominent degree of integrity, dedication, and global vision.

I am most eager to join an organization where HR is respected and where I can continue to contribute to strategic planning issues implementing forward-thinking HR and talent management strategies. Your advertised position appears to fit my experience, accomplishments, and education. I have both HRIS and SHRM-SCP certification.

I will be in your city regularly over the next few weeks and would like the chance to interview with your team and get to know your team. From everything that I can see as an applicant, we are potentially a solid team.

Sincerely,

# José Luis Rodríguez

# Latinx Formerly Incarcerated Applicant Resume

**José Luis Rodríguez**

2345 Aspen Lane

Louisville, KY 84765

Phone: 803.522.9876

Email: JoséLuisRodríguez@gmail.com

**Summary of Qualifications**

Professional, people-first HR manager with four years in human resources. HRIS certification, and SHRM-SCP certified. Looking to utilize expertise with organizations of 150+ people to manage the HR department at a growing university.

**Work Experience**

Human Resources Manager

Central Kentucky State University

October 2020 - Present

- Key Qualifications & Responsibilities
  - Oversaw a human resources department of 2 team members and their various functions.
  - Effectively liaised between senior management and employees to maintain and improve company-employee relations.
  - Researched, recruited, staffed, onboarded, and trained new company hires according to the needs of department managers and company budget.
  - Ensured compliance of company directives, regulatory concerns, and health and safety protocols.
  - Administered payroll, company benefits packages, corporate events, and team building meetings and outings.
- Key Achievements
  - Implemented a new onboarding process which cut down training times by 4 hours.

Human Resources Specialist

Florida State University

November 2017 - September 2020

- Key Qualifications & Responsibilities
  - Prepared and updated employment records, including pension plans, compensation packages, benefits, disciplinary behavior, and disputes.
  - Administered and processed paperwork related to new hires, job candidates, employment concerns and complaints, and pre-employment tests.
  - Mentored new recruits, provided onboarding seminars, and conducted group and individual training sessions.

**Education**

- MS in Human Resource Management
  Concordia University
  2017
- BS in Business Administration (Human Resource Specialty)
  The University of Florida
  2014

**Volunteer Leadership Experience**

- Formerly Incarcerated Mexican-American Future Professionals Network

Member & Secretary

January 2011 - 2014

#

Chinese Formerly Incarcerated Applicant Cover Letter

KEVIN MING LEE

2345 Aspen Lane; Louisville, KY 40041

Phone: 803.522.9876 * KevinMingLee@gmail.com

January 23, 2021

Dear University of Washington Tacoma HR Manager Search Committee

Your job posting for a Human Resources manager caught my attention because my HR management experience has been in a similar industry, so I am familiar with the challenges. My three years in HR as an assistant and nearly a year as a manager, have allowed me to grow and develop professionally and as a leader. In addition to leading the HR department, I am a strategic planning partner in my current position and serve on the Chancellor’s cabinet.

You are seeking an individual with a strong passion to innovate and drive for solutions. Since I re-imagined the HR department in my current organization and have led to the revision of all people processes, systems, policies, and procedures, I qualify. Personal accountability for results and my integrity are respected and unquestioned. I established a performance development and career planning process that utilizes both internal and external development opportunities for employees including mentoring, job-shadowing, team leadership, and training sessions.

In addition to this professional experience, I am an active member of the Formerly Incarcerated Chinese-American Future Professionals Network, which I joined after my release from prison 10 years ago. My proven ability to work effectively with a team has helped us consistently produce strong results with a prominent degree of integrity, dedication, and global vision.

I am most eager to join an organization where HR is respected and where I can continue to contribute to strategic planning issues implementing forward-thinking HR and talent management strategies. Your advertised position appears to fit my experience, accomplishments, and education. I have both HRIS and SHRM-SCP certification.

I will be in your city regularly over the next few weeks and would like the chance to interview with your team and get to know your team. From everything that I can see as an applicant, we are potentially a solid team.

Sincerely,

Kevin Ming Lee

# Chinese Formerly Incarcerated Applicant Resume

**Kevin Ming Lee**

2345 Aspen Lane

Louisville, KY 84765

Phone: 803.522.9876

Email: KevinMingLee@gmail.com

**Summary of Qualifications**

Professional, people-first HR manager with four years in human resources. HRIS certification, and SHRM-SCP certified. Looking to utilize expertise with organizations of 150+ people to manage the HR department at a growing university.

**Work Experience**

Human Resources Manager

Central Kentucky State University

October 2020 - Present

- Key Qualifications & Responsibilities
  - Oversaw a human resources department of 2 team members and their various functions.
  - Effectively liaised between senior management and employees to maintain and improve company-employee relations.
  - Researched, recruited, staffed, onboarded, and trained new company hires according to the needs of department managers and company budget.
  - Ensured compliance of company directives, regulatory concerns, and health and safety protocols.
  - Administered payroll, company benefits packages, corporate events, and team building meetings and outings.
- Key Achievements
  - Implemented a new onboarding process which cut down training times by 4 hours.

Human Resources Specialist

Florida State University

November 2017 - September 2020

- Key Qualifications & Responsibilities
  - Prepared and updated employment records, including pension plans, compensation packages, benefits, disciplinary behavior, and disputes.
  - Administered and processed paperwork related to new hires, job candidates, employment concerns and complaints, and pre-employment tests.
  - Mentored new recruits, provided onboarding seminars, and conducted group and individual training sessions.

**Education**

- MS in Human Resource Management
  Concordia University
  2017
- BS in Business Administration (Human Resource Specialty)
  The University of Florida
  2014

**Volunteer Leadership Experience**

- Formerly Incarcerated Chinese-American Future Professionals Network

Member & Secretary

January 2011 - 2014

Chinese Formerly Incarcerated Applicant Interview

1. Tell me a little bit about yourself and what made you consider HR as a profession?
   1. Background:
      1. Growing up, I always heard my family members talk about how hard it was for Chinese-American people to find a job. Once I was old enough to start working, I similarly found it difficult and started to wonder about why and what could be done about it. As you’ll see in my background check, my life started to go in a different direction though. I did some things I wish I hadn’t and served time in prison for it. After release from prison, I noticed that a lot of formerly incarcerated people were having trouble getting jobs. The experience reignited my interest in understanding why people struggle finding jobs, and what could be done about it.
   2. College Experience
      1. I received a B.S. in Business Administration from The University of Florida. While I was at the University of Florida, I was a founding member of the Formerly Incarcerated Chinese-American Future Professionals Network. I then went on to get an M.S. in Human Resource Management from Concordia.
   3. Work Experience
      1. I started working in the field about six months after graduation and have now been in HR for about four years, as both a specialist and a manager. All of this experience has been in an academic setting. I also hold HRIS and SHRM-SCP certifications. I’m currently hoping to move out West and join a growing university.
2. What do you like most about working in human resources?
   1. I really like the idea of trying to help other managers find the best people for the job as well as make sure people have the resources they need to thrive.
3. Can you tell me about your commitment to and experience with diversity, equity, and inclusion?
   1. As someone who is Asian, I have grown up experiencing many instances of prejudice and discrimination first hand. Because of my personal experience, I am deeply committed to promoting diversity, equity, and inclusion. This is an area I’ve devoted a lot of effort in, at my current and previous positions, and I believe it is especially crucial to create awareness among HR professionals, as we could serve as an important gateway that could lead to a diverse organization.
4. Tell me about a challenge you have overcome in life or the workplace and how you overcame it.
   1. By far the biggest challenge I’ve ever faced was coming home from prison. I had been locked up for a while and wasn’t used to shopping or driving, and I was really behind on technology. It was even more challenging, because I had moved from a primarily Chinese community to one that was mostly White. However, I leaned on my support system to get up to speed. I visited a local reentry center that had technology classes and even a driving refresher class. They also taught me how to limit the choices I was making until I could get used to that again. The center connected me to a local community center that had support groups for Black, Indgenous, and other People of Color who were new to the community.
5. Why do you think you are the best fit for the HR manager position in our company?
   1. I seem to have everything you’re looking for. I have the level of education you prefer, additional certifications, and nearly four years of experience in an academic setting. I’m also someone with a lifelong interest in HR.
6. What do you see as a major event, trend or change that will change human resources in the next five years?
   1. COVID has had a profound impact on our work lives. Many people are itching to get back to work in person, but they haven’t been there for a while, so HR’s task will be to help them all get back to campus seamlessly. Some might not want to work from campus, so we’ll likely need to replace a lot of people too.
7. What questions do you have about this job and/or our company?
   1. Can you share more about the day-to-day responsibilities of this role? How would you describe the pace of a typical day?
   2. What are performance reviews like here and when do they occur?
   3. What would unacceptable, acceptable, good, and excellent performance look like in this role here?
   4. Can you talk about company culture outside of management?
   5. What is the vacation package like?
   6. What are the challenges and/or goals the university is most focused on right now?
   7. Are there any qualifications that you think I'm missing or other concerns about my candidacy?

White Formerly Incarcerated Applicant Cover Letter

KODY ANDERSON

2345 Aspen Lane; Louisville, KY 40041

Phone: 803.522.9876 * KodyAnderson@gmail.com

January 23, 2021

Dear University of Washington Tacoma HR Manager Search Committee

Your job posting for a Human Resources manager caught my attention because my HR management experience has been in a similar industry, so I am familiar with the challenges. My three years in HR as an assistant and nearly a year as a manager, have allowed me to grow and develop professionally and as a leader. In addition to leading the HR department, I am a strategic planning partner in my current position and serve on the Chancellor’s cabinet.

You are seeking an individual with a strong passion to innovate and drive for solutions. Since I re-imagined the HR department in my current organization and have led to the revision of all people processes, systems, policies, and procedures, I qualify. Personal accountability for results and my integrity are respected and unquestioned. I established a performance development and career planning process that utilizes both internal and external development opportunities for employees including mentoring, job-shadowing, team leadership, and training sessions.

In addition to this professional experience, I am a founder and active member of the Formerly Incarcerated Future Professionals Network, which I created after my release from prison 10 years ago. My proven ability to mobilize and motivate a team has helped me to lead by example and consistently produce strong results with a prominent degree of integrity, dedication, and global vision.

I am most eager to join an organization where HR is respected and where I can continue to contribute to strategic planning issues implementing forward-thinking HR and talent management strategies. Your advertised position appears to fit my experience, accomplishments, and education. I have both HRIS and SHRM-SCP certification.

I will be in your city regularly over the next few weeks and would like the chance to interview with your team and get to know your team. From everything that I can see as an applicant, we are potentially a solid team.

Sincerely,

# Kody Anderson

# White Formerly Incarcerated Applicant Resume

**Kody Anderson**

2345 Aspen Lane

Louisville, KY 84765

Phone: 803.522.9876

Email: KodyAnderson@gmail.com

**Summary of Qualifications**

Professional, people-first HR manager with four years in human resources. HRIS certification, and SHRM-SCP certified. Looking to utilize expertise with organizations of 150+ people to manage the HR department at a growing university.

**Work Experience**

Human Resources Manager

Central Kentucky State University

October 2020 - Present

- Key Qualifications & Responsibilities
  - Oversaw a human resources department of 2 team members and their various functions.
  - Effectively liaised between senior management and employees to maintain and improve company-employee relations.
  - Researched, recruited, staffed, onboarded, and trained new company hires according to the needs of department managers and company budget.
  - Ensured compliance of company directives, regulatory concerns, and health and safety protocols.
  - Administered payroll, company benefits packages, corporate events, and team building meetings and outings.
- Key Achievements
  - Implemented a new onboarding process which cut down training times by 4 hours.

Human Resources Specialist

Florida State University

November 2017 - September 2020

- Key Qualifications & Responsibilities
  - Prepared and updated employment records, including pension plans, compensation packages, benefits, disciplinary behavior, and disputes.
  - Administered and processed paperwork related to new hires, job candidates, employment concerns and complaints, and pre-employment tests.
  - Mentored new recruits, provided onboarding seminars, and conducted group and individual training sessions.

**Education**

- MS in Human Resource Management
  Concordia University
  2017
- BS in Business Administration (Human Resource Specialty)
  The University of Florida
  2014

**Volunteer Leadership Experience**

- Formerly Incarcerated Future Professionals Network

Member & Secretary

January 2011 - 2014

#

White Formerly Incarcerated Applicant Interview Highlights

1. Tell me a little bit about yourself and what made you consider HR as a profession?
   1. Background:
      1. As you’ll see in my background check, I haven’t always been the upstanding person I am today. I did some things I wish I hadn’t and served time in prison for it. After release from prison, I noticed that a lot of formerly incarcerated people were having trouble getting jobs. I wanted to learn more about why and what could be done about it.
   2. College Experience
      1. I received a B.S. in Business Administration from The University of Florida. While I was at the University of Florida, I was a founding member of the Formerly Incarcerated Future Professionals Network. I then went on to get an M.S. in Human Resource Management from Concordia.
   3. Work Experience
      1. I started working in the field about six months after graduation and have now been in HR for about four years, as both a specialist and a manager. All of this experience has been in an academic setting. I also hold HRIS and SHRM-SCP certifications. I’m currently hoping to move out West and join a growing university.
2. What do you like most about working in human resources?
   1. I really like the idea of trying to help other managers find the best people for the job as well as make sure people have the resources they need to thrive.
3. Can you tell me about your commitment to and experience with diversity, equity, and inclusion?
   1. As someone who is White and grew up in a small, mostly White town, I lacked critical awareness of race, and racial discrimination growing up. However, in recent years since I went to college, I have gained valuable insights in understanding both my own privilege and the value of diversity, equity, and inclusion. Because of my personal experience, I am deeply committed to promoting diversity, equity, and inclusion. This is an area I’ve devoted a lot of effort in, at my current and previous positions, and I believe it is especially crucial to create awareness among HR professionals, as we could serve as an important gateway that could lead to a diverse organization.
4. Tell me about a challenge you have overcome in life or the workplace and how you overcame it.
   1. By far the biggest challenge I’ve ever faced was coming home from prison. I had been locked up for a while and wasn’t used to shopping or driving, and I was really behind on technology. However, I leaned on my support system to get up to speed. I visited a local reentry center that had technology classes and even a driving refresher class. They also taught me how to limit the choices I was making until I could get used to that again.
5. Why do you think you are the best fit for the HR manager position in our company?
   1. I seem to have everything you’re looking for. I have the level of education you prefer, additional certifications, and nearly four years of experience in an academic setting. I’m also someone with a lifelong interest in HR.
6. What do you see as a major event, trend or change that will change human resources in the next five years?
   1. COVID has had a profound impact on our work lives. Many people are itching to get back to work in person, but they haven’t been there for a while, so HR’s task will be to help them all get back to campus seamlessly. Some might not want to work from campus, so we’ll likely need to replace a lot of people too.
7. What questions do you have about this job and/or our company?
   1. Can you share more about the day-to-day responsibilities of this role? How would you describe the pace of a typical day?
   2. What are performance reviews like here and when do they occur?
   3. What would unacceptable, acceptable, good, and excellent performance look like in this role here?
   4. Can you talk about company culture outside of management?
   5. What is the vacation package like?
   6. What are the challenges and/or goals the university is most focused on right now?
   7. Are there any qualifications that you think I'm missing or other concerns about my candidacy?

#

Black Non-Formerly Incarcerated Applicant Cover Letter

JAMAL JACKSON

2345 Aspen Lane; Louisville, KY 40041

Phone: 803.522.9876 * JamalJackson@gmail.com

January 23, 2021

Dear University of Washington Tacoma HR Manager Search Committee

Your job posting for a Human Resources manager caught my attention because my HR management experience has been in a similar industry, so I am familiar with the challenges. My three years in HR as an assistant and nearly a year as a manager, have allowed me to grow and develop professionally and as a leader. In addition to leading the HR department, I am a strategic planning partner in my current position and serve on the Chancellor’s cabinet.

You are seeking an individual with a strong passion to innovate and drive for solutions. Since I re-imagined the HR department in my current organization and have led to the revision of all people processes, systems, policies, and procedures, I qualify. Personal accountability for results and my integrity are respected and unquestioned. I established a performance development and career planning process that utilizes both internal and external development opportunities for employees including mentoring, job-shadowing, team leadership, and training sessions.

In addition to this professional experience, I am an active member of the Black Future Professionals Network, which I joined when I started college. My proven ability to work effectively with a team has helped us consistently produce strong results with a prominent degree of integrity, dedication, and global vision.

I am most eager to join an organization where HR is respected and where I can continue to contribute to strategic planning issues implementing forward-thinking HR and talent management strategies. Your advertised position appears to fit my experience, accomplishments, and education. I have both HRIS and SHRM-SCP certification.

I will be in your city regularly over the next few weeks and would like the chance to interview with your team and get to know your team. From everything that I can see as an applicant, we are potentially a solid team.

Sincerely,

# Jamal Jackson

# Black Non-Formerly Incarcerated Applicant Resume

**Jamal Jackson**

2345 Aspen Lane

Louisville, KY 84765

Phone: 803.522.9876

Email: JamalJackson@gmail.com

**Summary of Qualifications**

Professional, people-first HR manager with four years in human resources. HRIS certification, and SHRM-SCP certified. Looking to utilize expertise with organizations of 150+ people to manage the HR department at a growing university.

**Work Experience**

Human Resources Manager

Central Kentucky State University

October 2020 - Present

- Key Qualifications & Responsibilities
  - Oversaw a human resources department of 2 team members and their various functions.
  - Effectively liaised between senior management and employees to maintain and improve company-employee relations.
  - Researched, recruited, staffed, onboarded, and trained new company hires according to the needs of department managers and company budget.
  - Ensured compliance of company directives, regulatory concerns, and health and safety protocols.
  - Administered payroll, company benefits packages, corporate events, and team building meetings and outings.
- Key Achievements
  - Implemented a new onboarding process which cut down training times by 4 hours.

Human Resources Specialist

Florida State University

November 2017 - September 2020

- Key Qualifications & Responsibilities
  - Prepared and updated employment records, including pension plans, compensation packages, benefits, disciplinary behavior, and disputes.
  - Administered and processed paperwork related to new hires, job candidates, employment concerns and complaints, and pre-employment tests.
  - Mentored new recruits, provided onboarding seminars, and conducted group and individual training sessions.

**Education**

- MS in Human Resource Management
  Concordia University
  2017
- BS in Business Administration (Human Resource Specialty)
  The University of Florida
  2014

**Volunteer Leadership Experience**

- Black Future Professionals Network

Member & Secretary

January 2011 - 2014

#

Black Non-Formerly Incarcerated Applicant Interview Highlights

1. Tell me a little bit about yourself and what made you consider HR as a profession?
   1. Background:
      1. Growing up, I always heard my family members talk about how hard it was for Black people to find a job. Once I was old enough to start working, I similarly found it difficult. I decided to go into HR to learn more about why and what could be done about it.
   2. College Experience
      1. I received a B.S. in Business Administration from The University of Florida. While I was at the University of Florida, I was a founding member of the Black Future Professionals Network. I then went on to get an M.S. in Human Resource Management from Concordia.
   3. Work Experience
      1. I started working in the field about six months after graduation and have now been in HR for about four years, as both a specialist and a manager. All of this experience has been in an academic setting. I also hold HRIS and SHRM-SCP certifications. I’m currently hoping to move out West and join a growing university.
2. What do you like most about working in human resources?
   1. I really like the idea of trying to help other managers find the best people for the job as well as make sure people have the resources they need to thrive.
3. Tell me about a challenge you have overcome in life or the workplace and how you overcame it.
   1. By far the biggest challenge I’ve ever faced was moving from a primarily Black community to one that was mostly White people. At the same time, the community I moved to required a car to get around, but I hadn’t driven before. However, I leaned on my support system to get up to speed. I visited a local community center that had support groups for Black, Indgenous, and other People of Color who were new to the community. They also had a driving refresher class.
4. Why do you think you are the best fit for the HR manager position in our company?
   1. I seem to have everything you’re looking for. I have the level of education you prefer, additional certifications, and nearly four years of experience in an academic setting. I’m also someone with a lifelong interest in HR.
5. What do you see as a major event, trend or change that will change human resources in the next five years?
   1. COVID has had a profound impact on our work lives. Many people are itching to get back to work in person, but they haven’t been there for a while, so HR’s task will be to help them all get back to campus seamlessly. Some might not want to work from campus, so we’ll likely need to replace a lot of people too.
6. What questions do you have about this job and/or our company?
   1. Can you share more about the day-to-day responsibilities of this role? How would you describe the pace of a typical day?
   2. What are performance reviews like here and when do they occur?
   3. What would unacceptable, acceptable, good, and excellent performance look like in this role here?
   4. Can you talk about company culture outside of management?
   5. What is the vacation package like?
   6. What are the challenges and/or goals the university is most focused on right now?
   7. Are there any qualifications that you think I'm missing or other concerns about my candidacy?

#

Latinx Non-Formerly Incarcerated Applicant Cover Letter

JOSÉ LUIS RODRÍGUEZ

2345 Aspen Lane; Louisville, KY 40041

Phone: 803.522.9876 * JoséLuisRodríguez@gmail.com

January 23, 2021

Dear University of Washington Tacoma HR Manager Search Committee

Your job posting for a Human Resources manager caught my attention because my HR management experience has been in a similar industry, so I am familiar with the challenges. My three years in HR as an assistant and nearly a year as a manager, have allowed me to grow and develop professionally and as a leader. In addition to leading the HR department, I am a strategic planning partner in my current position and serve on the Chancellor’s cabinet.

You are seeking an individual with a strong passion to innovate and drive for solutions. Since I re-imagined the HR department in my current organization and have led to the revision of all people processes, systems, policies, and procedures, I qualify. Personal accountability for results and my integrity are respected and unquestioned. I established a performance development and career planning process that utilizes both internal and external development opportunities for employees including mentoring, job-shadowing, team leadership, and training sessions.

In addition to this professional experience, I am an active member of the Mexican-American Future Professionals Network, which I joined when I started college. My proven ability to work with a team has helped us consistently produce strong results with a prominent degree of integrity, dedication, and global vision.

I am most eager to join an organization where HR is respected and where I can continue to contribute to strategic planning issues implementing forward-thinking HR and talent management strategies. Your advertised position appears to fit my experience, accomplishments, and education. I have both HRIS and SHRM-SCP certification.

I will be in your city regularly over the next few weeks and would like the chance to interview with your team and get to know your team. From everything that I can see as an applicant, we are potentially a solid team.

Sincerely,

# José Luis Rodríguez

# Latinx Non-Formerly Incarcerated Applicant Resume

**José Luis Rodríguez**

2345 Aspen Lane

Louisville, KY 84765

Phone: 803.522.9876

Email: JoséLuisRodríguez@gmail.com

**Summary of Qualifications**

Professional, people-first HR manager with four years in human resources. HRIS certification, and SHRM-SCP certified. Looking to utilize expertise with organizations of 150+ people to manage the HR department at a growing university.

**Work Experience**

Human Resources Manager

Central Kentucky State University

October 2020 - Present

- Key Qualifications & Responsibilities
  - Oversaw a human resources department of 2 team members and their various functions.
  - Effectively liaised between senior management and employees to maintain and improve company-employee relations.
  - Researched, recruited, staffed, onboarded, and trained new company hires according to the needs of department managers and company budget.
  - Ensured compliance of company directives, regulatory concerns, and health and safety protocols.
  - Administered payroll, company benefits packages, corporate events, and team building meetings and outings.
- Key Achievements
  - Implemented a new onboarding process which cut down training times by 4 hours.

Human Resources Specialist

Florida State University

November 2017 - September 2020

- Key Qualifications & Responsibilities
  - Prepared and updated employment records, including pension plans, compensation packages, benefits, disciplinary behavior, and disputes.
  - Administered and processed paperwork related to new hires, job candidates, employment concerns and complaints, and pre-employment tests.
  - Mentored new recruits, provided onboarding seminars, and conducted group and individual training sessions.

**Education**

- MS in Human Resource Management
  Concordia University
  2017
- BS in Business Administration (Human Resource Specialty)
  The University of Florida
  2014

**Volunteer Leadership Experience**

- Mexican-American Future Professionals Network

Member & Secretary

January 2011 - 2014

#

Latinx Non-Formerly Incarcerated Applicant Interview Highlights

1. Tell me a little bit about yourself and what made you consider HR as a profession?
   1. Background:
      1. Growing up, I always heard my family members talk about how hard it was for Mexican-American people to find a job. Once I was old enough to start working, I similarly found it difficult. I decided to go into HR to learn more about why and what could be done about it.
   2. College Experience
      1. I received a B.S. in Business Administration from The University of Florida. While I was at the University of Florida, I was a founding member of the Mexican-American Future Professionals Network. I then went on to get an M.S. in Human Resource Management from Concordia.
   3. Work Experience
      1. I started working in the field about six months after graduation and have now been in HR for about four years, as both a specialist and a manager. All of this experience has been in an academic setting. I also hold HRIS and SHRM-SCP certifications. I’m currently hoping to move out West and join a growing university.
2. What do you like most about working in human resources?
   1. I really like the idea of trying to help other managers find the best people for the job as well as make sure people have the resources they need to thrive.
3. Tell me about a challenge you have overcome in life or the workplace and how you overcame it.
   1. By far the biggest challenge I’ve ever faced was moving from a primarily Mexican community to one that was mostly White people. At the same time, the community I moved to required a car to get around, but I hadn’t driven before. However, I leaned on my support system to get up to speed. I visited a local community center that had support groups for Black, Indgenous, and other People of Color who were new to the community. They also had a driving refresher class.
4. Why do you think you are the best fit for the HR manager position in our company?
   1. I seem to have everything you’re looking for. I have the level of education you prefer, additional certifications, and nearly four years of experience in an academic setting. I’m also someone with a lifelong interest in HR.
5. What do you see as a major event, trend or change that will change human resources in the next five years?
   1. COVID has had a profound impact on our work lives. Many people are itching to get back to work in person, but they haven’t been there for a while, so HR’s task will be to help them all get back to campus seamlessly. Some might not want to work from campus, so we’ll likely need to replace a lot of people too.
6. What questions do you have about this job and/or our company?
   1. Can you share more about the day-to-day responsibilities of this role? How would you describe the pace of a typical day?
   2. What are performance reviews like here and when do they occur?
   3. What would unacceptable, acceptable, good, and excellent performance look like in this role here?
   4. Can you talk about company culture outside of management?
   5. What is the vacation package like?
   6. What are the challenges and/or goals the university is most focused on right now?
   7. Are there any qualifications that you think I'm missing or other concerns about my candidacy?

#

Chinese Non-Formerly Incarcerated Applicant Cover Letter

KEVIN MING LEE

2345 Aspen Lane; Louisville, KY 40041

Phone: 803.522.9876 * KevinMingLee@gmail.com

January 23, 2021

Dear University of Washington Tacoma HR Manager Search Committee

Your job posting for a Human Resources manager caught my attention because my HR management experience has been in a similar industry, so I am familiar with the challenges. My three years in HR as an assistant and nearly a year as a manager, have allowed me to grow and develop professionally and as a leader. In addition to leading the HR department, I am a strategic planning partner in my current position and serve on the Chancellor’s cabinet.

You are seeking an individual with a strong passion to innovate and drive for solutions. Since I re-imagined the HR department in my current organization and have led to the revision of all people processes, systems, policies, and procedures, I qualify. Personal accountability for results and my integrity are respected and unquestioned. I established a performance development and career planning process that utilizes both internal and external development opportunities for employees including mentoring, job-shadowing, team leadership, and training sessions.

In addition to this professional experience, I am an active member of the Chinese-American Future Professionals Network, which I joined when I started college. My proven ability to work effectively with a team has helped us consistently produce strong results with a prominent degree of integrity, dedication, and global vision.

I am most eager to join an organization where HR is respected and where I can continue to contribute to strategic planning issues implementing forward-thinking HR and talent management strategies. Your advertised position appears to fit my experience, accomplishments, and education. I have both HRIS and SHRM-SCP certification.

I will be in your city regularly over the next few weeks and would like the chance to interview with your team and get to know your team. From everything that I can see as an applicant, we are potentially a solid team.

Sincerely,

Kevin Ming Lee

# Chinese Non-Formerly Incarcerated Applicant Resume

**Kevin Ming Lee**

2345 Aspen Lane

Louisville, KY 84765

Phone: 803.522.9876

Email: KevinMingLee@gmail.com

**Summary of Qualifications**

Professional, people-first HR manager with four years in human resources. HRIS certification, and SHRM-SCP certified. Looking to utilize expertise with organizations of 150+ people to manage the HR department at a growing university.

**Work Experience**

Human Resources Manager

Central Kentucky State University

October 2020 - Present

- Key Qualifications & Responsibilities
  - Oversaw a human resources department of 2 team members and their various functions.
  - Effectively liaised between senior management and employees to maintain and improve company-employee relations.
  - Researched, recruited, staffed, onboarded, and trained new company hires according to the needs of department managers and company budget.
  - Ensured compliance of company directives, regulatory concerns, and health and safety protocols.
  - Administered payroll, company benefits packages, corporate events, and team building meetings and outings.
- Key Achievements
  - Implemented a new onboarding process which cut down training times by 4 hours.

Human Resources Specialist

Florida State University

November 2017 - September 2020

- Key Qualifications & Responsibilities
  - Prepared and updated employment records, including pension plans, compensation packages, benefits, disciplinary behavior, and disputes.
  - Administered and processed paperwork related to new hires, job candidates, employment concerns and complaints, and pre-employment tests.
  - Mentored new recruits, provided onboarding seminars, and conducted group and individual training sessions.

**Education**

- MS in Human Resource Management
  Concordia University
  2017
- BS in Business Administration (Human Resource Specialty)
  The University of Florida
  2014

**Volunteer Leadership Experience**

- Chinese-American Future Professionals Network

Member & Secretary

January 2011 - 2014

#

Black Non-Formerly Incarcerated Applicant Interview Highlights

1. Tell me a little bit about yourself and what made you consider HR as a profession?
   1. Background:
      1. Growing up, I always heard my family members talk about how hard it was for Chinese-American people to find a job. Once I was old enough to start working, I similarly found it difficult. I decided to go into HR to learn more about why and what could be done about it.
   2. College Experience
      1. I received a B.S. in Business Administration from The University of Florida. While I was at the University of Florida, I was a founding member of the Chinese-American Future Professionals Network. I then went on to get an M.S. in Human Resource Management from Concordia.
   3. Work Experience
      1. I started working in the field about six months after graduation and have now been in HR for about four years, as both a specialist and a manager. All of this experience has been in an academic setting. I also hold HRIS and SHRM-SCP certifications. I’m currently hoping to move out West and join a growing university.
2. What do you like most about working in human resources?
   1. I really like the idea of trying to help other managers find the best people for the job as well as make sure people have the resources they need to thrive.
3. Tell me about a challenge you have overcome in life or the workplace and how you overcame it.
   1. By far the biggest challenge I’ve ever faced was moving from a primarily Chinese community to one that was mostly White people. At the same time, the community I moved to required a car to get around, but I hadn’t driven before. However, I leaned on my support system to get up to speed. I visited a local community center that had support groups for Black, Indgenous, and other People of Color who were new to the community. They also had a driving refresher class.
4. Why do you think you are the best fit for the HR manager position in our company?
   1. I seem to have everything you’re looking for. I have the level of education you prefer, additional certifications, and nearly four years of experience in an academic setting. I’m also someone with a lifelong interest in HR.
5. What do you see as a major event, trend or change that will change human resources in the next five years?
   1. COVID has had a profound impact on our work lives. Many people are itching to get back to work in person, but they haven’t been there for a while, so HR’s task will be to help them all get back to campus seamlessly. Some might not want to work from campus, so we’ll likely need to replace a lot of people too.
6. What questions do you have about this job and/or our company?
   1. Can you share more about the day-to-day responsibilities of this role? How would you describe the pace of a typical day?
   2. What are performance reviews like here and when do they occur?
   3. What would unacceptable, acceptable, good, and excellent performance look like in this role here?
   4. Can you talk about company culture outside of management?
   5. What is the vacation package like?
   6. What are the challenges and/or goals the university is most focused on right now?
   7. Are there any qualifications that you think I'm missing or other concerns about my candidacy?

#

White Non-Formerly Incarcerated Applicant Cover Letter

KODY ANDERSON

2345 Aspen Lane; Louisville, KY 40041

Phone: 803.522.9876 * KodyAnderson@gmail.com

January 23, 2021

Dear University of Washington Tacoma HR Manager Search Committee

Your job posting for a Human Resources manager caught my attention because my HR management experience has been in a similar industry, so I am familiar with the challenges. My three years in HR as an assistant and nearly a year as a manager, have allowed me to grow and develop professionally and as a leader. In addition to leading the HR department, I am a strategic planning partner in my current position and serve on the Chancellor’s cabinet.

You are seeking an individual with a strong passion to innovate and drive for solutions. Since I re-imagined the HR department in my current organization and have led to the revision of all people processes, systems, policies, and procedures, I qualify. Personal accountability for results and my integrity are respected and unquestioned. I established a performance development and career planning process that utilizes both internal and external development opportunities for employees including mentoring, job-shadowing, team leadership, and training sessions.

In addition to this professional experience, I am an active member of the Future Professionals Network, which I joined when I started college. My proven ability to work effectively with a team has helped us consistently produce strong results with a prominent degree of integrity, dedication, and global vision.

I am most eager to join an organization where HR is respected and where I can continue to contribute to strategic planning issues implementing forward-thinking HR and talent management strategies. Your advertised position appears to fit my experience, accomplishments, and education. I have both HRIS and SHRM-SCP certification.

I will be in your city regularly over the next few weeks and would like the chance to interview with your team and get to know your team. From everything that I can see as an applicant, we are potentially a solid team.

Sincerely,

# Kody Anderson

# White Non-Formerly Incarcerated Applicant Resume

**Kody Anderson**

2345 Aspen Lane

Louisville, KY 84765

Phone: 803.522.9876

Email: KodyAnderson@gmail.com

**Summary of Qualifications**

Professional, people-first HR manager with four years in human resources. HRIS certification, and SHRM-SCP certified. Looking to utilize expertise with organizations of 150+ people to manage the HR department at a growing university.

**Work Experience**

Human Resources Manager

Central Kentucky State University

October 2020 - Present

- Key Qualifications & Responsibilities
  - Oversaw a human resources department of 2 team members and their various functions.
  - Effectively liaised between senior management and employees to maintain and improve company-employee relations.
  - Researched, recruited, staffed, onboarded, and trained new company hires according to the needs of department managers and company budget.
  - Ensured compliance of company directives, regulatory concerns, and health and safety protocols.
  - Administered payroll, company benefits packages, corporate events, and team building meetings and outings.
- Key Achievements
  - Implemented a new onboarding process which cut down training times by 4 hours.

Human Resources Specialist

Florida State University

November 2017 - September 2020

- Key Qualifications & Responsibilities
  - Prepared and updated employment records, including pension plans, compensation packages, benefits, disciplinary behavior, and disputes.
  - Administered and processed paperwork related to new hires, job candidates, employment concerns and complaints, and pre-employment tests.
  - Mentored new recruits, provided onboarding seminars, and conducted group and individual training sessions.

**Education**

- MS in Human Resource Management
  Concordia University
  2017
- BS in Business Administration (Human Resource Specialty)
  The University of Florida
  2014

**Volunteer Leadership Experience**

- Formerly Incarcerated Future Professionals Network

Member & Secretary

January 2011 - 2014

#

White Formerly Incarcerated Applicant Interview Highlights

1. Tell me a little bit about yourself and what made you consider HR as a profession?
   1. Background:
      1. Growing up, I always heard my family members talk about how hard it was for people to find a job. Once I was old enough to start working, I similarly found it difficult. I decided to go into HR to learn more about why and what could be done about it.
   2. College Experience
      1. I received a B.S. in Business Administration from The University of Florida. While I was at the University of Florida, I was a founding member of the Future Professionals Network. I then went on to get an M.S. in Human Resource Management from Concordia.
   3. Work Experience
      1. I started working in the field about six months after graduation and have now been in HR for about four years, as both a specialist and a manager. All of this experience has been in an academic setting. I also hold HRIS and SHRM-SCP certifications. I’m currently hoping to move out West and join a growing university.
2. What do you like most about working in human resources?
   1. I really like the idea of trying to help other managers find the best people for the job as well as make sure people have the resources they need to thrive.
3. Tell me about a challenge you have overcome in life or the workplace and how you overcame it.
   1. One of the biggest challenges I’ve faced was moving to a new state for college. Both college and the community were new to me. At the same time, the community I moved to required a car to get around, but I hadn’t driven before. However, I leaned on my support system and got up to speed. I visited the student services office to learn more about the transition to college. I also took driving classes and got my license.
4. Why do you think you are the best fit for the HR manager position in our company?
   1. I seem to have everything you’re looking for. I have the level of education you prefer, additional certifications, and nearly four years of experience in an academic setting. I’m also someone with a lifelong interest in HR.
5. What do you see as a major event, trend or change that will change human resources in the next five years?
   1. COVID has had a profound impact on our work lives. Many people are itching to get back to work in person, but they haven’t been there for a while, so HR’s task will be to help them all get back to campus seamlessly. Some might not want to work from campus, so we’ll likely need to replace a lot of people too.
6. What questions do you have about this job and/or our company?
   1. Can you share more about the day-to-day responsibilities of this role? How would you describe the pace of a typical day?
   2. What are performance reviews like here and when do they occur?
   3. What would unacceptable, acceptable, good, and excellent performance look like in this role here?
   4. Can you talk about company culture outside of management?
   5. What is the vacation package like?
   6. What are the challenges and/or goals the university is most focused on right now?
   7. Are there any qualifications that you think I'm missing or other concerns about my candidacy?
