# Supplementary material for: Incarceration history and ethnic bias in hiring perceptions: An experimental test of intersectional bias & psychological mechanisms
Source: PLoS One. 2023 Jan 17;18(1):e0280397. doi: 10.1371/journal.pone.0280397 (PMC9844837; doi:10.1371/journal.pone.0280397)
Supplement: S4 Appendix — (DOCX) [file pone.0280397.s004.docx]

# Appendix D - Measures of Morality, Sociability/Warmth, Competence

### Please indicate on a 7-point scale, from 1 (*not at all*) and 7 (*very much*), the extent to which each of the following traits characterizes the job candidate you just read about.

1. Sincere
2. Honest
3. Trustworthy
4. Friendly
5. Warm
6. Likeable
7. Intelligent
8. Competent
9. Skillful
