# Supplementary material for: Incarceration history and ethnic bias in hiring perceptions: An experimental test of intersectional bias & psychological mechanisms
Source: PLoS One. 2023 Jan 17;18(1):e0280397. doi: 10.1371/journal.pone.0280397 (PMC9844837; doi:10.1371/journal.pone.0280397)
Supplement: S5 Appendix — (DOCX) [file pone.0280397.s005.docx]

# Appendix E - Perceived hireability and Hiring Decision

Hireability

1. How likely would you be willing to hire this candidate?
2. To what extent is this a top-notch candidate?
3. Is it likely that this candidate will make an effective director?
4. How excellent is this candidate based on this letter and resume?
