# Supplementary material for: Incarceration history and ethnic bias in hiring perceptions: An experimental test of intersectional bias & psychological mechanisms
Source: PLoS One. 2023 Jan 17;18(1):e0280397. doi: 10.1371/journal.pone.0280397 (PMC9844837; doi:10.1371/journal.pone.0280397)
Supplement: S6 Appendix — (DOCX) [file pone.0280397.s006.docx]

# Appendix F - Manipulation Checks

Manipulation Checks

1. What was the ethnicity of the job applicant?
   1. Asian
   2. Mexican
   3. Black
   4. White
   5. Other
2. Has the job applicant been to prison or otherwise incarcerated?
   1. Yes, the applicant has been to prison or otherwise incarcerated
   2. No, the applicant has not been to prison or otherwise incarcerated
   3. I don't know if the applicant has been to prison or otherwise incarcerated
3. (if previously incarcerated). What was the person incarcerated for?
   1. A “white collar” crime. (crimes related to fraud, generally associated with harm to institutions such as business and government but also inclusive of financial harms to people). For example, public corruption, health care fraud, mortgage fraud, securities fraud, and money laundering.
   2. A “blue collar” crime. (crimes that are not related to finances or corruption, generally associated with physical and psychological harms against people rather than institutions). For example, drug crimes, theft, burglary, assault, and murder.
