# Supplementary material for: Incarceration history and ethnic bias in hiring perceptions: An experimental test of intersectional bias & psychological mechanisms
Source: PLoS One. 2023 Jan 17;18(1):e0280397. doi: 10.1371/journal.pone.0280397 (PMC9844837; doi:10.1371/journal.pone.0280397)
Supplement: S7 Appendix — (DOCX) [file pone.0280397.s007.docx]

# Appendix G - Demographics Survey

Demographics Survey

1. How old are you?
   1. Enter
2. What industry do you work in?
   1. Business and Financial Operations Occupations
   2. Computer and Mathematical Occupations
   3. Architecture and Engineering Occupations
   4. Life, Physical, and Social Science Occupations
   5. Community and Social Service Occupations
   6. Legal Occupations
   7. Educational Instruction and Library Occupations
   8. Arts, Design, Entertainment, Sports, and Media Occupations
   9. Healthcare Practitioners and Technical Occupations
   10. Healthcare Support Occupations
   11. Protective Service Occupations
   12. Food Preparation and Serving Related Occupations
   13. Building and Grounds Cleaning and Maintenance Occupations
   14. Personal Care and Service Occupations
   15. Sales and Related Occupations
   16. Office and Administrative Support Occupations
   17. Farming, Fishing, and Forestry Occupations
   18. Construction and Extraction Occupations
   19. Installation, Maintenance, and Repair Occupations
   20. Production Occupations
   21. Transportation and Material Moving Occupations
3. What is the size of your current departments/units that you oversee or manage?
4. How many years have you been in a role where you are responsible for hiring employees?
5. What is your gender?
   1. Enter
6. What ethnicity do you identify most with?
   1. Enter
