# Supplementary material for: Incarceration history and ethnic bias in hiring perceptions: An experimental test of intersectional bias & psychological mechanisms
Source: PLoS One. 2023 Jan 17;18(1):e0280397. doi: 10.1371/journal.pone.0280397 (PMC9844837; doi:10.1371/journal.pone.0280397)
Supplement: Attachment — Submitted filename: PLOS ONE Review.docx [file pone.0280397.s008.docx]

The authors are proposing to carry out a much-needed study about the mechanisms underlying discriminatory processes in hiring by race/ethnicity and incarceration history. We know stark disparities exist in employment/hireability by both of these characteristics, but we know much less about why. My comments are meant to strengthen the proposed design and connect these authors to criminological and sociological work that might bolster the contribution of this study. My comments are as follows:

- The discussion justifying the use of “intergroup threat” seems thin. I think in the context of employers, it should also focus on “risk” with perceived threat as a core component, but for liability and safety reasons. Sugie and colleagues (2020) might help the authors think this point through a bit more. Also, see Vuolo et al. (2017) who discuss how labor market conditions (increased competition) also shape the extent to which marginalized groups are discriminated against, with recessions exacerbating racial inequalities in callbacks for jobs. While I think it's fine to keep the measures that the authors have for threat in Appendix J, they might also consider including indicators about liability/safety that are specific to the work context. So, the authors could include “customers and other employees,” in addition to their broader threat items, which may provide more information about the mechanisms specific to the context of employment. For instance, employers may not see incarcerated people as threats more broadly, but view them as potential liabilities to their business, nonetheless. Authors should also see Reich 2017 and DeWitt and Denver 2020 for similar study designs and conceptual frameworks.
  - Sugie, N. F., Zatz, N. D., & Augustine, D. (2020). Employer aversion to criminal records: An experimental study of mechanisms. Criminology, 58(1), 5–34.
  - Vuolo, M., Uggen, C., & Lageson, S. (2017). Race, Recession, and Social Closure in the Low-Wage Labor Market: Experimental and Observational Evidence. Research in the Sociology of Work, 30, 141–183.
  - Reich, S. E. (2017). An exception to the rule: Belief in redeemability, desistance signals, and the employer’s decision to hire a job applicant with a criminal record. Journal of Offender Rehabilitation, 56(2), 110–136.
  - DeWitt, S. E., & Denver, M. (2020). Criminal Records, Positive Employment Credentials, and Race. Journal of Research in Crime and Delinquency, 57(3), 333–368.
- On pages 12-13, the authors suggest that the position will be for a human resources manager, but do not provide any context as to *why* that particular position is a good case for understanding the role of incarceration and race in perceived hireability. Additionally, the authors provide little information about how the job ads were constructed (thinking about external validity).
- The authors may want to add additional questions to Appendix M about the size of respondents’ departments/units in which they were hiring managers and the number of years they’ve been in these roles.
- Why signal incarceration through being a founder and president of an organization for formerly incarcerated people? Does that signal something additional about incarcerated applicants that may not be externally valid in the same way that signaling incarceration through something common/more realistic like their education or work experience might? I would like the authors to contextualize this decision and the implications that it might have on their results, especially when attempting to test concepts like morality, warmth, and competence as mechanisms (e.g., Does this make the results potentially more conservative?)
- The authors do not justify the name selection. I would suggest using Gaddis (2017) as a potential starting point for name selection to ensure race/ethnicity is signaled and not other characteristics like class. This point also makes me wonder if the authors should be using “Americanized” Asian names vs. traditional names, or both to account for employer assumptions/stereotypes about English fluency and language.
  - Gaddis, S. (2017). How Black Are Lakisha and Jamal? Racial Perceptions from Names Used in Correspondence Audit Studies. Sociological Science, 4, 469–489.
